# Filamentous Algae for Wastewater Circularity: A Review of Wastewater Treatment, Resource Recovery, and Biorefinery Opportunities

**DOI:** 10.3390/microorganisms14081702

**Published:** 2026-08-03

**Authors:** Songqi Yang, Li Cao, Chenyang Wei, Xi Luo, Haoyang Li, Tangyun Zhang, Shenghui Yang, Guanghong Luo

**Affiliations:** 1 Gansu Microalgae Technology Innovation Center, Hexi University, Zhangye 734000, China; 2 College of Agriculture and Ecological Engineering, Hexi University, Zhangye 734000, China; 3 College of Life Science and Engineering, Hexi University, Zhangye 734000, China

**Keywords:** resource recovery, algal biorefinery, emerging contaminant removal, circular bioeconomy, biofertilizers

## Abstract

Wastewater treatment is undergoing a transition from pollutant removal toward resource recovery, creating opportunities to integrate environmental remediation with circular bioeconomy principles. Filamentous algae have attracted increasing attention as multifunctional biological platforms because their attached growth habit facilitates biomass harvesting while supporting nutrient recovery and biomass valorization. This review synthesizes current knowledge on the roles of filamentous algae in wastewater treatment, with emphasis on nutrient and contaminant removal, biomass production, and the generation of bioenergy, biofertilizers, aquafeeds, and cellulose-based biomaterials. It highlights how filamentous algae differ from conventional suspended microalgae through improved biomass retention, simpler harvesting, and compatibility with attached-growth systems such as algal turf scrubbers and biofilm reactors. The review also examines the ecological interactions between filamentous algae and associated microbial communities that underpin nutrient cycling and treatment performance. Beyond wastewater treatment, it critically evaluates the opportunities and challenges associated with downstream biomass valorization, including biofuel production and the recovery of high-value products within integrated biorefinery frameworks. In addition, the review discusses the principal barriers to large-scale implementation, including limited field-scale validation, variability in biomass quality, contaminant safety, downstream processing requirements, regulatory uncertainty, and the need for comprehensive techno-economic and environmental assessments. Finally, it highlights emerging research directions involving systems biology, advanced process monitoring, artificial intelligence-assisted process control, and integrated biorefinery concepts that may support future development. By integrating biological, engineering, and sustainability perspectives, this review provides a comprehensive framework for understanding the potential of filamentous algae to support resilient wastewater treatment systems and accelerate the transition toward circular resource management.

## 1. Introduction

The incessant pressures of global urbanization, industrialization, and climate change have exposed fundamental limitations in conventional wastewater treatment systems, which were historically designed for pollutant removal rather than resource recovery [1]. Traditional activated sludge systems are effective at reducing organic load and nutrients but are energy-intensive and inherently linear due to the dissipation of valuable carbon, nitrogen, and phosphorus rather than reintegrating them into productive cycles [2]. The reorganization of wastewater treatment from a cost center into a resource-generating platform because the potential for recovering valuable resources such as energy, nutrients, and water from wastewater is becoming increasingly important for sustainable development [3]. The shifting towards a circular economy approach will enable wastewater treatment plants to not only improve environmental outcomes but also create economic opportunities through resource recovery [4]. Algae-driven wastewater treatment has gained renewed attention due to its ability to couple contaminant removal with biomass generation, CO_2_ sequestration, and downstream valorization [5]. Filamentous algae have demonstrated effective removal of nutrients, organic matter, heavy metals, and selected emerging contaminants, while photosynthetic oxygen production can support aerobic microbial processes and reduce external aeration requirements in algal–bacterial treatment systems [6]. This dual functionality of improving water quality and reducing the energy consumption of treatment systems aligns closely with circular economy principles, where resources are used efficiently and waste is minimized through closed-loop processes [7]. However, despite decades of research on unicellular microalgae systems, several persistent bottlenecks still hinder large-scale implementation, such as the high cost of cultivation, inefficient harvesting methods, and limited understanding of microbial interactions within microalgae cultivation systems. Overcoming these challenges will be crucial for maximizing the potential of microalgae in sustainable wastewater treatment and resource recovery processes [8].

This limitation unicellular microalgae have triggers interest towards the engineering of filamentous algae such as *Cladophora* sp. *Spirogyra* sp. and *Oedogonium* sp., as they have shown promise in addressing some of the current bottlenecks in microalgae cultivation [9]. This is attributed to the fact that filamentous algae have macroscopic, thread-like morphologies that enable straightforward harvesting through simple physical methods such as screening or filtration. This intrinsic property directly addresses one of the most critical cost barriers in algal biotechnology, where harvesting can account for a substantial fraction of total operational expenditure [10]. Moreover, filamentous algae demonstrate enhanced resistance to predation, improved settleability, and the capacity to form stable mats or biofilms, which can be leveraged for process intensification and reactor simplification [6]. Recent studies have demonstrated a shift from viewing filamentous algae merely as a niche alternative to a transformative platform for wastewater circularity. Studies have shown that filamentous strains can achieve nutrient removal efficiencies exceeding 90% while maintaining high biomass productivity under various operational regimes [11]. Furthermore, their ability to decouple hydraulic retention time (HRT) from solid retention time (SRT) offers new degrees of freedom in reactor design, enabling higher throughput and improved process control compared to conventional microalgal systems. These advantages are particularly relevant for large-scale applications, where land footprint and operational simplicity are critical constraints [12]. The strategic importance of filamentous algae lies in their compatibility with integrated biorefinery concepts. Algal biomass derived from wastewater can be processed into a wide array of value-added products, including biofuels, animal feed, fertilizers, and bioplastics, thereby closing material loops and enhancing economic viability. Filamentous algae are also emerging tools for utilization on a multi-stage treatment platform due to their ability to complement other biological processes such as anaerobic digestion or bacterial granulation [13].

Despite the advantage of filamentous algae for wastewater circularity, the current body of literature remains fragmented and constrained by narrow experimental scopes or system-specific analyses. Most reviews have primarily focused on either microalgal consortia or general algal applications while overlooking the distinct ecological, physiological, and engineering attributes of filamentous algae. Many dedicated reviews on filamentous systems have highlighted their potential for nutrient removal, biofuel production, and biomass valorization. However, there is still a need for more comprehensive and integrated studies that consider the full range of benefits and challenges associated with filamentous algae in wastewater treatment systems [14]. Also, there are limited studies synthesizing how filamentous algae can be systematically remodeled from passive biomass formers into dynamic bioresources for achieving multiple, concurrent objectives such as nutrient recovery, energy production, and greenhouse gas mitigation [15]. Another critical gap lies in the translation of laboratory-scale findings to real-world applications because the high efficiencies obtained in controlled experiments have persisting uncertainties resulting from system robustness under fluctuating environmental conditions [16]. The re-thinking of filamentous algae is therefore pertinent to address these challenges and optimize the potential of this resource for sustainable biotechnological applications. Previous reviews have examined important aspects of filamentous algae, including nutrient removal, biomass production, bioenergy generation, and cultivation strategies, but most have focused on one or a limited number of applications. This review provides a comprehensive synthesis of the diverse functional roles of filamentous algae within wastewater circularity by integrating contaminant removal, biomass valorization, and the production of value-added bioproducts into a single framework. In addition, it combines biological and engineering perspectives to illustrate how filamentous algae can be strategically incorporated into multi-objective wastewater treatment systems. Beyond summarizing recent advances, this review critically discusses techno-economic, operational, and regulatory challenges that remain important barriers to commercialization and proposes future research directions involving emerging technologies, including artificial intelligence, systems biology, and integrated biorefinery concepts. By identifying current knowledge gaps and research priorities, this review aims to support the transition of filamentous algae from laboratory research toward practical wastewater treatment and resource recovery applications [17].

## 2. Taxonomic and Functional Aspects of Filamentous Algae Beyond Nitrogen and Phosphorus Removal

Filamentous algae are morphologically and phylogenetically diverse, representing multicellular or filament-forming photosynthetic organisms whose closest relatives include both unicellular and multicellular taxa. These organisms span multiple evolutionary lineages, including green algae (Chlorophyta), cyanobacteria (Cyanophyta), and heterokont groups such as Xanthophyceae [18,19]. Although many studies have focused more on green filamentous taxa such as *Cladophora* sp., *Spirogyra* sp., *Oedogonium* sp., and *Ulothrix* sp., due to their ecological importance and adaptability to variable nutrient regimes and ease of harvesting [20], a narrow emphasis on nitrogen (N) and phosphorus (P) removal has often overshadowed the broader taxonomic–functional relationships that underpin the ecological success and technological potential of these organisms. A deeper understanding of these aspects is essential for retooling filamentous algae as dynamic, multi-objective agents in wastewater circularity. This alludes to the fact that practical selection of a target species for large-scale systems like Filamentous Algae Nutrient Scrubbers (FANS) extends far beyond simple N and P uptake rates, involving a complex interplay of competitive dominance, seasonal resilience, and biomass quality. A mesocosm-scale study by Hariz et al. (2023) [21] comparing monocultures of *Cladophora* sp., *Oedogonium* sp., *Rhizoclonium* sp., and *Spirogyra* sp. under ambient summer and winter conditions found that *Oedogonium* sp. was the best-performing species overall, not only because of its high biomass productivity and nitrate removal rates but critically due to its ability to maintain dominance over non-target species and tolerate seasonal variations in temperature and light [22]. This demonstrates that a species’ functional role, its capacity to sustain a stable monoculture, and its resistance to invasion are key determinants of its technological viability, a factor that is often overlooked when screening solely for nutrient removal efficiency [21]. The filamentous morphology of filamentous algae has evolved independently (convergently) across multiple algal clades, allowing for diverse metabolic capabilities and ecological advantages in nutrient-rich and hydrodynamically variable environments [22]. For example, *Oedogonium* sp. and *Cladophora* sp. exhibit uniseriate filaments with high surface-to-volume ratios, facilitating efficient mass transfer of nutrients and gases. Their cell walls are often composed of cellulose and hemicellulose together with pectic polysaccharides, depending on the genus and developmental stage, which provide structural rigidity while allowing for flexibility under shear stress [23], whereas filamentous cyanobacteria such as *Oscillatoria* sp. and *Anabaena* sp. possess peptidoglycan-based cell walls surrounded by an outer membrane characteristic of Gram-negative bacteria and can form trichomes, with genera such as *Anabaena* developing specialized cells (heterocysts) for nitrogen fixation [24,25]. Interspecific variations also influence the metabolic capabilities of filamentous algae beyond conventional nutrient removal. This alludes to the fact that functional attributes such as the capacity for carbon capture and transformation through differences in photosynthetic carbon-concentrating mechanisms, carbon allocation, and storage metabolism can vary significantly among different species of filamentous algae [26,27]. Most filamentous algae, such as *Spirogyra* sp., *Cladophora* sp., and *Oedogonium* sp., exhibit distinct physiological strategies shaped by their evolutionary lineages. For instance, *Cladophora* sp. often tolerate high light and temperature fluctuations and show efficient carbon fixation in eutrophic waters; though they frequently occur together in polluted waters, these are largely distinct physiological advantages and not causally linked [28]. While *Spirogyra* sp. typically thrives in freshwater habitats, including quiet ponds, shallow lakes, canals, and slow-moving streams, with different species occurring across oligotrophic to eutrophic conditions [29], these differences directly impact carbon capture rates, metabolic byproducts like extracellular polymeric substances (EPS), and pathways for organic carbon transformation. Some taxa release substantial dissolved organic carbon through EPS and other exudates that stimulate heterotrophic microbial communities, whereas others preferentially store fixed carbon as starch, lipids, or structural carbohydrates depending on species and environmental conditions, offering potential for biofuel feedstocks [27,29]. Also, nitrogen and sulfur assimilation pathways vary because certain filamentous cyanobacteria, such as *Anabaena* sp. and *Nostoc* sp., have specialized cells called heterocysts that provide a micro-oxic environment required for nitrogenase activity, thereby modifying carbon–nitrogen metabolic coupling between vegetative cells and heterocysts [30].

The functional attributes of filamentous algae beyond N and P removal thus warrant further investigation to fully understand their potential benefits in wastewater treatment processes. This is attributed to their high efficiency in the assimilation of inorganic carbon such as CO_2_ and bicarbonate through photosynthesis, often mediated by carbon-concentrating mechanisms (CCMs) that enhance inorganic carbon acquisition under carbon-limited conditions, contributing to both biomass production and pH modulation in wastewater systems [31,32]. The fixing of inorganic carbon into organic matter has the potential to reduce net CO_2_ emissions from wastewater treatment systems, depending on biomass harvesting, downstream utilization, and life-cycle system boundaries, while generating valuable feedstocks for bioenergy or biofertilizer production [33]. Moreover, their mat-forming growth habit facilitates the entrapment of suspended solids and heavy metals through physical filtration, sedimentation enhancement, and extracellular polymeric substance (EPS)-mediated aggregation, thereby enhancing effluent clarity and reducing toxicity while potentially minimizing the requirement for additional chemical coagulants or adsorbents [34]. Filamentous structures also generate oxygen-rich surface layers and oxygen-limited internal microenvironments within algal mats, supporting diverse microbial consortia and potentially improving the degradation of complex organic pollutants through syntrophic interactions [35,36,37]. The EPS produced by filamentous algae has been shown to play a crucial role in enhancing the removal of contaminants by acting as reactive interfaces that facilitate sorption and mediate interactions with co-existing microbial communities. These EPS matrices are chemically complex, containing polysaccharides, proteins, and functional groups capable of binding metals and organic compounds, thereby extending the functional scope of filamentous systems [38]. The EPS has also been shown to bind selected emerging contaminants such as pharmaceuticals, including antibiotics and anti-inflammatory drugs or endocrine-disrupting compounds, offering an important but still underexplored bioremediation pathway [39,40]. These multifunctional roles thus indicate that the integration of filamentous algae into engineered treatment systems could transform conventional wastewater management into a resource-recovery model. These knowledge gaps highlight the need for future research to prioritize the characterization of species-specific capabilities, optimization of cultivation parameters, techno-economic analysis, and life-cycle assessment to unlock their full potential in sustainable water treatment [6,32].

The interspecific differences also play a critical role in shaping the ability of filamentous algae to remove heavy metals such as cadmium, lead, chromium, and arsenic, which are commonly present in industrial wastewaters. For example, *Cladophora* sp. exhibits a high affinity for cadmium and lead, demonstrating removal efficiencies of 88.78% and 94.85%, respectively, while showing a significantly lower affinity for arsenic, with only 23.10% removal efficiency in comparative studies [41]. This is due to their thick, multilayered cell walls rich in polysaccharides and functional groups such as carboxyl, amino, and hydroxyl moieties. These cell walls also contain abundant uronic acids that further enhance metal binding. These groups facilitate biosorption through ion exchange and complexation, enabling rapid metal sequestration even at low concentrations [42]. In contrast, filamentous algae such as *Anabaena* sp., *Oscillatoria* sp., *Phormidium* sp., and *Spirogyra* sp. have been shown to reduce Cr(VI) by 70.96, 80.64, 76.12, and 74.83%, respectively, in tannery effluent through the process of bio-reduction and bio-sorption [43]. Table 1 provides a comprehensive overview of the various filamentous algal species and their potential uses in wastewater treatment, highlighting key differences in functional traits that may impact their effectiveness in different applications.

**Figure 1:**
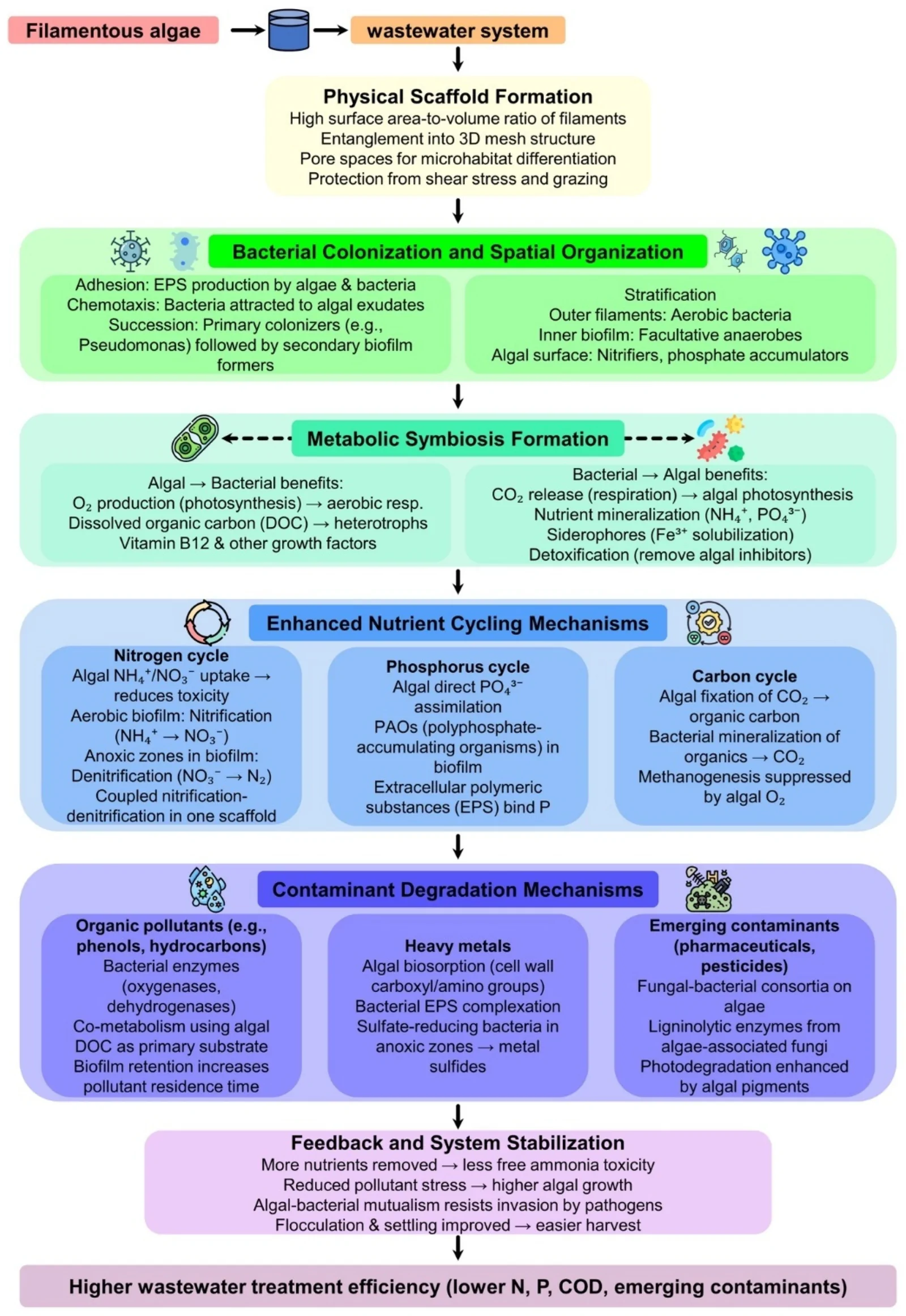
Illustrating the mechanistic insight into how filamentous algae reshape microbial community structure and interactions in wastewater systems. The figure shows the key biological, physical, and chemical mechanisms at each stage of the process, highlighting the intricate relationship between filamentous algae and microbial communities.

Interspecific variations also influence metal tolerance thresholds, regeneration potential, and post-harvest metal recovery options. Some filamentous green algae can be desorbed and reused for multiple cycles, whereas others offer direct valorization as metal-enriched biomass for resource recovery, metal recycling, or catalyst synthesis [66,67]. The understanding of these taxon-specific mechanisms is crucial for selecting appropriate algal strains for targeted bioremediation and for engineering consortia capable of treating complex industrial wastewater streams. Another critical aspect of taxonomic differences affecting the functional trait of filamentous algae is their effect on their ability to regulate oxygen dynamics and redox conditions within wastewater systems. This is because filamentous algae can generate oxygen in situ during photosynthesis, leading to the partial or, under favorable operating conditions, substantial reduction in the need for mechanical aeration [68,69]. This ability to produce oxygen can also promote the growth of aerobic bacteria through the creation of steep oxygen and redox gradients within biofilms and algal mats. These gradients support diverse microbial processes, including nitrification, denitrification, and even anaerobic pathways such as methanogenesis in deeper layers, thereby creating spatial heterogeneity that enables simultaneous multi-process treatment within a single reactor configuration. Figure 1 shows how filamentous algae trigger the reshaping of microbial community structure and interactions by providing a scaffold for bacterial colonization, thereby fostering symbiotic relationships that enhance nutrient cycling and contaminant degradation within wastewater systems [70,71].

## 3. Novel Application of Filamentous Algae Outside Pond Remediation

The need for novel applications of filamentous algae beyond traditional pond remediation is driven by the demand for sustainable, low-energy wastewater treatment systems. This has stimulated the development of several engineered applications of filamentous algae beyond conventional pond-based remediation, including continuous-flow algal turf scrubbers (ATS), heavy metal and emerging contaminant removal, algal–bacterial–fungal biofilm systems, and treatment under cold-climate or high-ammonia conditions [72]. The algal turf scrubber (ATS) is an attached-growth technology inspired by natural stream periphyton and represents one of the most established engineered applications of filamentous algae [73]. Filamentous algae have also attracted increasing interest for the removal of heavy metals and emerging contaminants because of their high surface area and EPS, although the removal of Per- and polyfluoroalkyl substances (PFAS) remains an emerging area of investigation and is currently attributed primarily to adsorption rather than biodegradation. Furthermore, integrated algal–bacterial–fungal biofilm systems are being explored for their complementary microbial functions in contaminant removal. Emerging evidence also supports the application of psychrotolerant filamentous algae in low-temperature wastewater treatment and the use of adapted biofilm consortia for wastewaters containing elevated ammonia concentrations. These developments highlight the expanding potential of filamentous algae as versatile platforms for municipal, industrial, and agricultural wastewater treatment, with their underlying mechanisms and specific applications discussed in subsequent sections [74].

### 3.1. The Application of Filamentous Microalgae in Continuous Flow Algal Turf Scrubbers

ATS are emerging technological platforms developed for algae-based wastewater treatment for harnessing the ecological and functional advantages of filamentous microalgae. This system is conceptualized to mimic the productivity and self-purification mechanisms of natural stream ecosystems by employing shallow, continuously flowing wastewater over inclined surfaces where attached algal communities develop as dense turfs [75]. ATS has shown promising results in terms of nutrient removal and biomass production due to the suitability of filamentous algae [55]. The suitability of filamentous algae in ATS applications can be attributed to their strong attachment capacity, rapid growth, efficient nutrient assimilation, and ease of biomass recovery. These characteristics position filamentous algae-based ATS as a critical interface between wastewater remediation and circular bioresource generation [76]. The operational principle of ATS is relatively simple yet biologically sophisticated because wastewater flows across a textured substrate under controlled hydraulic and light conditions, which enables filamentous algae and associated microbial consortia to colonize the surface [77]. As the algal turf matures, nutrients and contaminants are assimilated into biomass while oxygen is generated through photosynthesis, leading to the periodic harvesting of the algal mat, which maintains productivity and the removal of assimilated pollutants from the system [78]. ATS systems exploit attached growth, unlike suspended microalgal cultures, so they overcome many of the limitations associated with biomass separation and reactor instability [79]. The predominance of filamentous taxa within these systems is not incidental, but rather due to their morphology and ecological strategies that are inherently compatible with the hydraulic and physical demands of continuous-flow operation [80]. Filamentous microalgae such as *Klebsormidium* sp., *Stigeoclonium* spp., *Spirogyra* sp. and *Ulothrix* sp. have been shown to be well adapted to ATS environments because their elongated multicellular structures facilitate strong surface adhesion and resistance to hydraulic shear. These traits allow stable biofilm formation even under fluctuating flow regimes, reducing biomass washout and improving operational reliability. In many ATS systems, filamentous formations rapidly outcompete unicellular algae due to their superior light acquisition and spatial occupation strategies. Their ability to form dense mats also contributes to the increased retention time of nutrients and organic matter within the biofilm matrix, enhancing the overall treatment efficiency [11].

**Figure 2:**
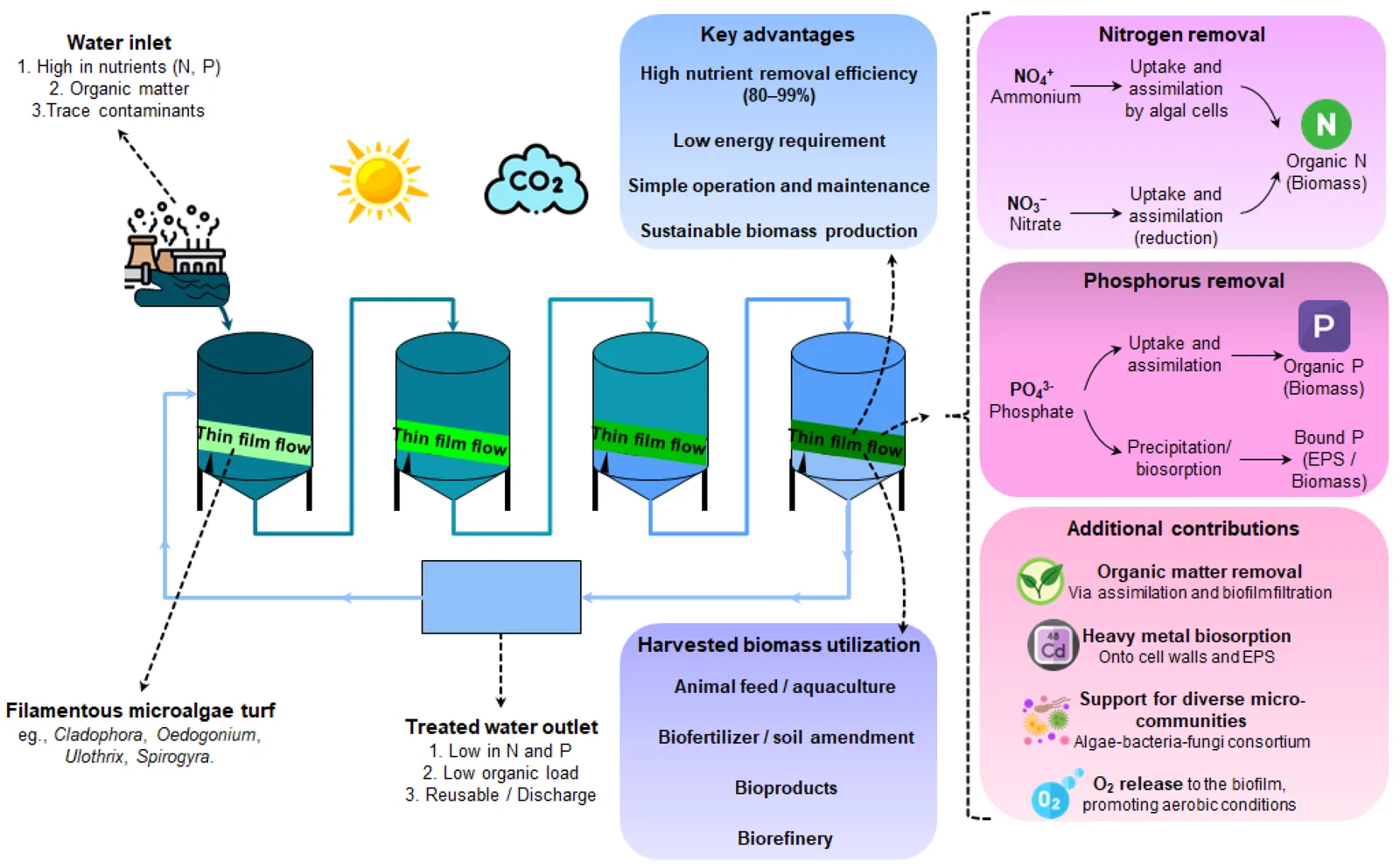
Role of filamentous algal turf in wastewater circularity. The figure explains how the oxygenation capacity of filamentous algal turfs maintains DO levels without external aeration, enabling wastewater circularity (nutrient recovery, water reuse, and biomass valorization).

The critical advantage of filamentous algae-based ATS systems is their high nutrient removal performance with nitrogen and phosphorus removal efficiencies exceeding 90–99% and total nitrogen up to 80–100% within a 7 day period under optimized conditions [81]. However, the significance of ATS extends beyond nutrient polishing because the continuous-flow configuration enables simultaneous removal of suspended solids, organic carbon, pathogens, and trace contaminants through a combination of biological uptake, adsorption, photodegradation, and microbial interactions. This multifunctionality reflects the dynamic ecological structure of algal turfs, where algae, bacteria, fungi, and protozoa coexist within highly structured biofilms [82]. This packed coupling between autotrophic and heterotrophic processes enhances treatment stability and aligns ATS technology with low-carbon wastewater management strategies, as shown in Figure 2. The key feature of continuous-flow ATS systems is hydrodynamics, which strongly influences the dominance and productivity of filamentous algae. This alludes to the fact that flow velocity, turbulence, and pulsed water delivery affect nutrient diffusion, light penetration, and biomass detachment [13]. Filamentous algae are particularly advantageous in this context because their flexible structures can withstand periodic shear while maintaining a high photosynthetic surface area. This allows them to outcompete other algae species and dominate in turbulent environments because their ability to adapt to changing conditions gives them a competitive advantage [83]. The design of reactor parameters such as slope angle, substrate roughness, flow periodicity, and hydraulic loading rate can greatly influence the growth and productivity of filamentous algae in turbulent environments. The optimization of these parameters creates conditions that promote the dominance of filamentous algae and maximize biomass production [84].

The choice of substrate is another critical factor in ATS performance because studies exploring a variety of materials, including plastic meshes, geotextiles, nylon screens, and roughened polymer surfaces, have shown that different substrates can impact the attachment and growth of filamentous algae differently [85]. This is attributed to the ability of filamentous algae to adhere to and colonize on different surfaces based on their texture, roughness, and material composition. Over time, the developing turf creates a complex three-dimensional matrix that enhances microbial diversity and functional redundancy. This structural complexity contributes to system resilience under variable environmental conditions and wastewater compositions. A major advantage of ATS systems compared to suspended algal cultivation is the simplicity of biomass harvesting. The dense mat of filamentous algae in ATS systems allows for easy mechanical harvesting, reducing the energy and cost associated with biomass collection [86]. Additionally, the harvested biomass can be easily separated from the water, making it more economically feasible and operationally practical at larger scales. Moreover, regular harvesting stimulates new growth and promotes continuous nutrient uptake, enhancing the overall efficiency of the system. This efficient nutrient removal also helps to prevent eutrophication in receiving water bodies, making ATS systems a sustainable solution for wastewater treatment [87]. The harvested biomass is an important component of wastewater circularity because the filamentous algal biomass generated in ATS systems can be used for various applications such as biofuel production, animal feed, or even as a soil conditioner. This closed-loop approach not only improves the overall sustainability of wastewater treatment but also contributes to the development of a circular economy [88]. *Spirogyra* sp. have demonstrated favorable carbohydrate profiles for bioethanol production, while mixed algal turfs have shown potential for methane generation through anaerobic digestion [89]. The integration of ATS with downstream biorefineries thus transforms wastewater treatment into a resource recovery platform, enhancing both environmental and economic sustainability [88].

Despite their advantages, ATS systems face several key limitations that require attention for broader implementation. For example, the process of mechanical harvesting using rotating brushes and scrapers requires careful optimization to avoid substrate damage or incomplete biomass removal, both of which reduce treatment efficiency. Harvesting frequency also presents a critical trade-off that maximizes nutrient removal but increases costs, while less frequent harvesting risks biomass overgrowth, self-shading, and detachment. The high moisture content of harvested biomass (85–95%) makes dewatering a major energy barrier, accounting for up to 30% of operational costs [90]. Additionally, harvested biomass is a complex mixture of algae, bacteria, and particulates, which may contain heavy metals or pathogens that restrict its use in feed or soil applications [91]. Other limitations include land requirements; seasonal light and temperature variability tend to affect productivity due to treatment inconsistency caused by environmental factors and the suitability of urban land for ATS systems [92]. The ATS system is also vulnerable to grazers such as snails, insect larvae, and pathogens, which can reduce the overall biomass yield and efficiency, particularly due to the fact that high-flow events may cause biomass detachment and washout. The ability of ATS to remove recalcitrant organic pollutants and pathogens is also limited by the need for regular maintenance and monitoring to ensure optimal performance. Additionally, the cost of implementing and maintaining ATS systems is also a barrier to widespread adoption, because it requires significant financial investment and expertise. Addressing these limitations requires continued research into strain selection, process optimization, harvesting technologies, and integrated biorefinery approaches [93].

### 3.2. Role of Filamentous Algae in Heavy Metal and Emerging Contaminant Removal

**Figure 3:**
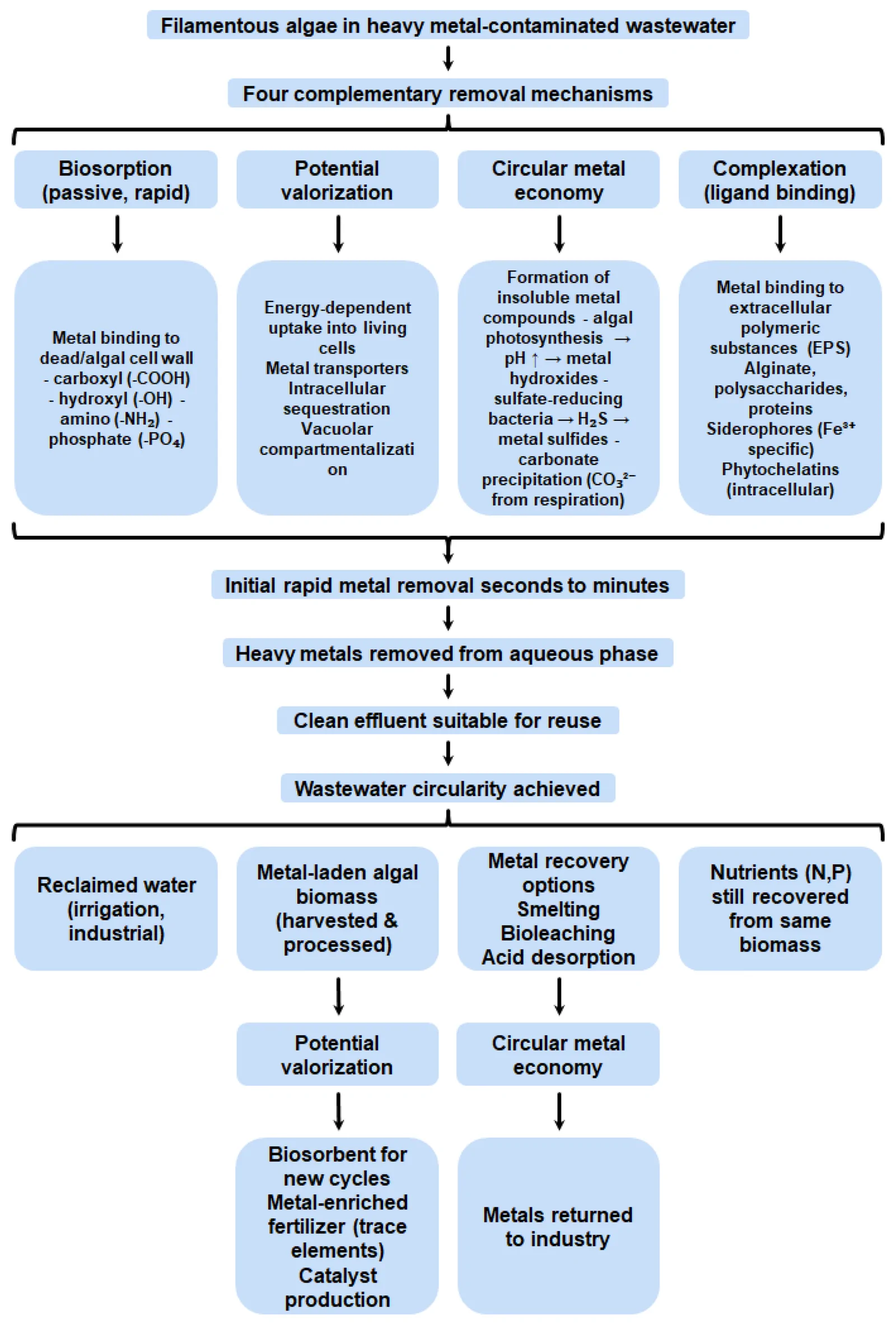
Depicting the mechanistic insight into how filamentous algae remove heavy metals from wastewater through biosorption, bioaccumulation, precipitation, and complexation and how this leads to wastewater circularity.

The evolution of wastewater treatment toward circular and multi-objective frameworks has triggered an increase in attention directed to contaminants that extend beyond conventional nutrient pollution [94]. The incessant industrialization, pharmaceutical consumption, agricultural intensification, and urban activities have led to the widespread occurrence of heavy metals and emerging contaminants (ECs) in aquatic environments. These pollutants, including pharmaceuticals, personal care products, endocrine-disrupting compounds, dyes, pesticides, microplastics, and antibiotic resistance determinants, are often poorly removed by conventional wastewater treatment processes [95]. Filamentous algae is an emerging biological tool that is capable of simultaneously supporting contaminant removal, biomass generation, and resource recovery [96]. The capacity of filamentous algae to remove heavy metals is among their most extensively investigated non-nutrient functions, with metals such as cadmium (Cd), lead (Pb), chromium (Cr), copper (Cu), zinc (Zn), arsenic (As), mercury (Hg), and nickel (Ni) being the most extensively studied [97]. Unlike organic pollutants, these metals cannot be mineralized due to their persistent environmental and non-biodegradable nature and toxicity even at low concentrations. So it must, therefore, be immobilized, transformed, or recovered for safe disposal or potential reuse. This makes filamentous algae a promising tool for addressing this challenge through a combination of biosorption, bioaccumulation, precipitation, and complexation mechanisms [98]. The mechanism used by filamentous algae in the removal of heavy metals involves the binding of metal ions to the cell wall through functional groups such as carboxyl, hydroxyl, and amino groups, as shown in Figure 3. Biosorption is generally considered the dominant and fastest mechanism involved in metal removal by filamentous algae. The cell walls of filamentous algae such as *Cladophora* sp., *Spirogyra* sp., *Oedogonium* sp., and *Pithophora* sp. contain abundant functional groups such as hydroxyl, carboxyl, amino, phosphate, and sulfate moieties that interact electrostatically or chemically with dissolved metal ions [99]. The high surface-to-volume ratio of filamentous structures enhances the availability of binding sites, while EPS secreted by algal mats further increases sorption capacity. This makes filamentous algae particularly effective biosorbents in industrial wastewaters containing mixed metal contaminants [100].

Interspecific differences within filamentous algae strongly influences metal uptake efficiency, with some species, such as green filamentous algae, exhibiting high affinity for divalent metal ions due to their cellulose-rich cell walls and polysaccharide composition [67]. For example, *Cladophora glomerata* has demonstrated substantial adsorption capacities for lead and cadmium [101], while *Spirogyra* species have shown efficient chromium and copper sequestration [102]. Several studies have documented the capacity of filamentous cyanobacteria to immobilize arsenic and mercury through sulfhydryl-rich extracellular matrices. For instance, *Nostoc linckia* has been shown to effectively remove chromium through biosorption and complexation with thiol-containing EPS components [103], while *Halomicronema* sp. produce EPS that act as a protective barrier, reducing metal bioavailability and toxicity by sequestering them outside the cell. These differences underscore the importance of species selection when designing algae-based remediation systems [104,105]. Filamentous algae can also actively accumulate metals intracellularly through metabolic transport processes. Once internalized, metals may be compartmentalized into vacuoles, bound to metallothioneins or phytochelatins, or transformed into less toxic forms. This intracellular sequestration contributes to detoxification and enables algae to tolerate relatively high metal concentrations [106]. However, excessive accumulation can inhibit photosynthesis, disrupt membrane integrity, and reduce biomass productivity. Consequently, understanding metal tolerance thresholds is critical for maintaining system performance under contaminated conditions. One of the major advantages of filamentous algae over unicellular microalgae in metal remediation is the ease of biomass recovery. Since metals become associated with the algal biomass, efficient harvesting is essential to prevent re-release into treated water. The macroscopic and entangled morphology of filamentous algae allows simple mechanical removal through screening or scraping, significantly reducing operational costs compared to centrifugation-dependent microalgal systems. Moreover, harvested metal-laden biomass can potentially be processed for metal recovery, biochar production, or safe immobilization, thereby contributing to circular resource management strategies [107].

Filamentous algae are surfacing as a tool in the removal of ECs, which are a highly diverse group of pollutants that are continuously introduced into wastewater systems through domestic, industrial, agricultural, and healthcare activities [108]. Pharmaceuticals such as antibiotics, analgesics, and hormones are among the most studied ECs due to their persistence and ecological impacts [109]. Filamentous algae contribute to EC removal through several complementary mechanisms, including bio adsorption, biodegradation, photo transformation, and co-metabolic interactions with associated microbial communities [110]. Adsorption onto algal cell walls and EPS matrices is particularly important for hydrophobic compounds, while intracellular uptake may occur for smaller or more lipophilic molecules. In some cases, enzymatic systems within algae facilitate the transformation of pharmaceuticals into less toxic metabolites. For instance, oxidative enzymes and reactive oxygen species generated during photosynthesis can contribute to the degradation of dyes, phenols, and endocrine-disrupting compounds. Several studies have demonstrated the effectiveness of filamentous algae in removing specific ECs. For example, *Cladophora glomerata* has shown significant removal of atrazine from eutrophic agricultural rivers through bioconcentration [111]. *Pithophora* sp. has been shown to remove the reactive amoxicillin antibiotic from aqueous solutions through biosorption [112], whereas *Ulva mutabilis,* in association with bacteria, reduced the concentration of endocrine disruptors, including bisphenol A, estradiol, and ethinylestradiol, by over 98% to below the detection limit [113]. The photosynthetic activity of filamentous algae also indirectly enhances EC removal by altering physicochemical conditions within treatment systems. Elevated dissolved oxygen concentrations stimulate aerobic bacterial degradation pathways, while pH fluctuations influence contaminant speciation and sorption behavior [114]. Additionally, exposure to sunlight in open algal systems promotes photodegradation of certain compounds, further contributing to removal efficiency. These synergistic interactions highlight the importance of considering filamentous algae not as isolated organisms but as central components of complex ecological treatment networks [115]. Antibiotics and antibiotic resistance genes (ARGs) have emerged as critical environmental concerns due to their role in the proliferation of antimicrobial resistance. Recent studies suggest that filamentous algal systems may contribute to ARG attenuation by reducing antibiotic concentrations and modifying microbial community dynamics. Although the exact mechanisms remain poorly understood, several pathways have been proposed, including adsorption of extracellular DNA, oxidative-stress-induced degradation, and competitive suppression of resistant bacterial populations. This area remains relatively underexplored but represents an important frontier in algae-based wastewater research [116]. Filamentous algae have also shown promise in the remediation of synthetic dyes and industrial organic pollutants. The textile and dyeing industries discharge large quantities of recalcitrant colorants that are often toxic and resistant to biodegradation [117]. Studies involving *Spirogyra* and *Cladophora* species have demonstrated substantial dye removal through adsorption and enzymatic degradation mechanisms [118,119]. In some systems, algal–bacterial consortia further enhance degradation efficiency through complementary metabolic pathways. Thus, supporting the importance of the integration of filamentous algae into hybrid treatment systems targeting complex industrial effluents. Another emerging area involves the interaction between filamentous algae and microplastics. Although research is still limited, evidence suggests that filamentous algal mats can trap microplastic particles through physical entanglement and biofilm formation. This process may facilitate downstream separation and reduce the mobility of microplastics in aquatic systems. However, the long-term ecological implications of algal–microplastic interactions remain uncertain, particularly regarding contaminant transfer within food webs and biomass valorization pathways [120].

### 3.3. Novel Insight into Filamentous Algal–Bacterial–Fungal Consortia on Moving Beds

**Figure 4:**
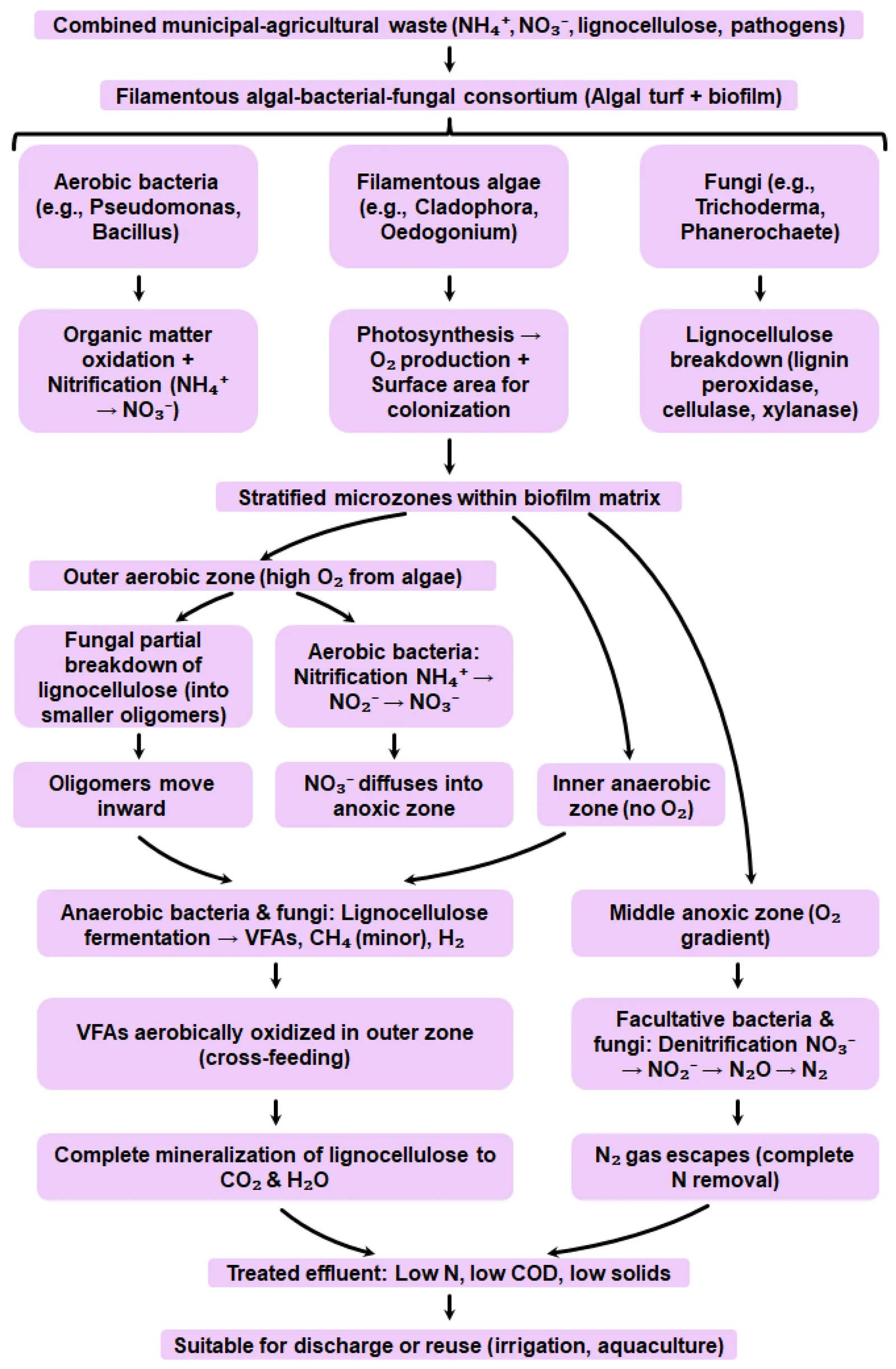
This figure depicts the mechanistic flow of how the combination of filamentous algae with bacteria and fungi consortium achieves simultaneous nitrification, denitrification, and lignocellulose breakdown that enables the treatment of combined municipal–agricultural wastewater (high in ammonia, organics, and crop residues).

Moving bed systems incorporating filamentous algae are a sustainable and efficient way to treat wastewater by utilizing the natural filtration capabilities of filamentous algae. These systems can help remove nutrients and pollutants from water, improving the overall water quality and reducing environmental impact [121]. These systems consist of reactors containing suspended carrier media that provide protected surfaces for biofilm attachment and microbial growth under continuous mixing or aeration. Carrier materials such as polyethylene, polypropylene, polyurethane, and bio-based polymers are selected based on their surface characteristics, density, durability, and cost to promote stable biofilm formation. Despite their advantages, continuous mixing increases energy demand, prolonged carrier abrasion may generate microplastics, and limited light penetration can restrict algal photosynthesis in deeper reactor zones. These limitations can be mitigated through optimized carrier loading, intermittent mixing, appropriate reactor depth, and supplementary lighting where required [122]. Building on these engineering considerations, increasing attention has focused on multispecies consortia involving filamentous algae, bacteria, and fungi, as shown in Figure 4. These systems employ attached biofilms on suspended carriers, where complementary interactions among algae, bacteria, and fungi improve nutrient transformation, contaminant degradation, and process stability under variable wastewater conditions. Consequently, moving-bed algal–bacterial–fungal systems have emerged as promising platforms for municipal, industrial, and agricultural wastewater treatment [123]. For example, Cao et al. demonstrated that filamentous algae, particularly *Leptolyngbya* sp. and *Geitlerinema* sp., acted as a backbone by tightly binding microbial aggregates and forming a strong core within algae–bacteria granules. This prevented disintegration that commonly affected aerobic granular sludge with integrity coefficient (IC) measurements that indicated that algae–bacteria granules had much lower values (0.12–0.24) compared to aerobic granular sludge (0.19–0.48), indicating superior stability. This signifies that filamentous algae provided more attachment points for bacteria, leading to increased EPS production, which further enhanced particle strength and overall stability [124]. This shows that filamentous algae play a foundational role within these consortia due to their morphology and ecological engineering capacity. Their elongated multicellular structures facilitate rapid colonization of carrier surfaces and create a three-dimensional matrix that supports the attachment of bacterial and fungal hyphae [125]. This physical scaffolding enhances biofilm thickness, structural cohesion, and resistance to shear stress generated by moving bed hydrodynamics. Filamentous algae such as *Cladophora* sp., *Oedogonium* sp. and *Spirogyra* sp. have demonstrated strong adaptability to attached-growth environments, where their filamentous architecture promotes efficient nutrient interception and light capture [55]. The inclusion of fungi introduces additional functional complexity to moving bed consortia because filamentous fungi contribute extensive hyphal networks that intertwine with algal filaments and bacterial aggregates, enhancing biofilm stability and mechanical resilience. Fungal hyphae also facilitate substrate penetration and transport within dense biofilms, improving nutrient accessibility and reducing diffusional limitations [126]. More importantly, fungi possess diverse extracellular enzymatic systems capable of degrading recalcitrant organic pollutants, including dyes, phenols, pharmaceuticals, and lignocellulosic compounds. This enzymatic versatility complements the photosynthetic and assimilatory functions of filamentous algae, enabling broader contaminant removal within a single integrated system [127].

The interactions between algae, bacteria, and fungi within moving beds are highly synergistic and metabolically interconnected. In this association, bacterial respiration generates carbon dioxide that fuels algal growth, leading to the establishment of a mutually beneficial carbon–oxygen exchange loop. Fungi contribute further by mineralizing complex organic substrates into simpler compounds that can be assimilated by both algae and bacteria. This trophic integration enhances overall treatment efficiency and creates a more resilient microbial ecosystem capable of adapting to fluctuating wastewater conditions [128].

Hydrodynamic movement within these systems plays a critical role in shaping the consortium structure and functionality, since continuous carrier motion improves mixing and mass transfer while preventing excessive biomass accumulation and localized anaerobic zones. Unlike static biofilm systems, moving beds expose microbial communities to periodic shear forces that selectively favor strongly attached and structurally integrated organisms [129]. Filamentous algae and fungi are particularly advantageous under these conditions because their intertwined growth forms an enhanced attachment strength and reduce biofilm detachment. At the same time, moderate shear promotes biofilm renewal and prevents excessive self-shading, thereby maintaining photosynthetic activity [130]. One of the most significant novel insights emerging from recent studies is the recognition that filamentous algal–bacterial–fungal consortia function not merely as additive microbial assemblages but as highly coordinated ecological networks [131]. Metabolic cross-feeding, signaling interactions, and spatial niche differentiation contribute to emergent properties that exceed the capabilities of individual organisms. For example, bacterial communities associated with algal biofilms often exhibit enhanced nitrification and denitrification activity due to oxygen gradients generated by photosynthesis. Simultaneously, fungal-mediated degradation of refractory compounds can release dissolved organic carbon that stimulates bacterial metabolism and secondary algal growth. These interactions create dynamic microenvironments that support simultaneous carbon, nitrogen, phosphorus, and contaminant removal [132].

Recent molecular and metagenomic studies demonstrated that microbial diversity within moving bed consortia strongly influences system stability and resistance to environmental stress. Diverse communities exhibit greater functional redundancy, enabling treatment performance to be maintained despite fluctuations in temperature, hydraulic loading, or pollutant composition [133]. Filamentous algae contribute significantly to this resilience by stabilizing biofilm architecture and modulating microenvironmental conditions such as pH and dissolved oxygen. Some studies also suggest that algae-derived EPS facilitate microbial communication and protect associated microorganisms from toxic shocks or oxidative stress [134]. Moving bed consortia incorporated with filamentous algae have shown increasing promise for the treatment of emerging contaminants and industrial effluents. The combination of fungal oxidative enzymes, bacterial biodegradation pathways, and algal biosorption mechanisms enables simultaneous attenuation of pharmaceuticals, dyes, heavy metals, and endocrine-disrupting compounds [135]. For example, the application of fungal laccases and peroxidases in textile wastewater treatment triggers the degradation of dye molecules while algal biomass adsorbs residual contaminants and assimilates released nutrients. Similarly, in pharmaceutical wastewater, bacterial transformation pathways can work synergistically with algal photodegradation processes to improve overall contaminant removal efficiency [136]. Another important dimension of filamentous algal–bacterial–fungal consortia is their contribution to greenhouse gas mitigation and energy efficiency [137]. The biomass generated from filamentous algal–bacterial–fungal consortia also offer significant opportunities for resource recovery and biorefinery integration. Mixed microbial biomass harvested from moving beds can be processed into biogas, biofertilizers, biochar, or platform chemicals depending on its composition and contamination profile. Fungal components may enhance anaerobic digestibility by partially degrading complex polysaccharides, while algal carbohydrates contribute to methane or bioethanol production potential [138].

### 3.4. Utilization of Filamentous Algae in Cold-Climate and High-Ammonia Systems

The expanding application of filamentous algae in wastewater treatment has increasingly highlighted the need to evaluate system performance under environmental and operational extremes. Among the most challenging conditions are cold-climate environments and wastewaters characterized by elevated ammonia concentrations [44]. Conventional biological treatment systems often experience reduced efficiency under such conditions due to inhibited microbial metabolism, poor nitrification, increased energy demand, and process instability [139]. In contrast, recent studies have suggested that filamentous algae possess several adaptive and functional traits that may enable them to perform effectively in these challenging environments. These emerging insights are particularly important as wastewater infrastructure faces growing climatic variability, stricter nutrient regulations, and increasing industrial and agricultural nitrogen loads [140].

Cold-climate conditions present a substantial challenge to wastewater treatment due to multiple interacting environmental stressors, including reduced wastewater temperatures, shortened photoperiods, lower solar irradiance, increased water viscosity, and, in some regions, ice formation. These factors collectively suppress microbial metabolism, reduce algal photosynthetic activity, and impair mass transfer processes, thereby decreasing the overall treatment efficiency [141,142]. Conventional activated sludge systems are especially vulnerable since nitrifying bacteria are highly temperature-sensitive and require substantial aeration energy to maintain treatment performance during winter conditions [143]. In algae-based systems, cold-climate limitations are often associated with reduced photosynthetic activity and slower biomass productivity. However, filamentous algae have demonstrated a remarkable degree of ecological adaptability in temperate and cold aquatic ecosystems, suggesting potential advantages over many unicellular microalgae under cold-climate conditions [144]. Filamentous algae exhibit considerable tolerance to cold environments, with many psychrotolerant taxa maintaining photosynthetic activity at temperatures close to freezing (approximately 2–4 °C), whereas mesophilic species generally exhibit reduced but measurable metabolic activity at temperatures below approximately 10 °C [145]. Cold-adapted filamentous genera, including *Ulothrix* sp., *Cladophora* sp., *Microspora* sp., and *Oedogonium* sp., are frequently reported in streams, rivers, lakes, and wastewater stabilization systems during winter and early spring, where low temperatures and reduced light availability favor their persistence over many competing algal taxa [44,146,147].

One notable insight from recent studies is that cold-climate may selectively favor filamentous growth forms over unicellular algae because filamentous structures provide advantages in nutrient interception, surface attachment, and resistance to hydraulic disturbances common in cold-weather wastewater systems [148]. In open channels and algal turf scrubbers (ATS), filamentous mats can maintain stable biofilms despite fluctuating flow conditions and seasonal temperature shifts. Their dense morphology also creates microenvironments within biofilms that buffer cells against sudden thermal changes, enhancing ecological resilience [55]. The interaction between light availability and temperature is particularly important in cold-climate algal systems. Winter conditions are often associated with shorter photoperiods and lower solar intensity, which can constrain photosynthesis. However, filamentous algae possess high pigment plasticity and can adapt chlorophyll composition to optimize light utilization under suboptimal conditions [149]. Some studies report that cold-adapted filamentous microalgae maintain relatively high photosynthetic efficiency at low irradiance, enabling sustained nutrient uptake during winter operation. This ability is especially advantageous in high-latitude regions where conventional biological treatment processes experience seasonal performance declines [150]. The reactor configuration is also critical in the support of filamentous algae under cold conditions. Different reactor designs can impact the growth and distribution of filamentous algae, with some configurations providing more favorable conditions for their development. For example, attached-growth systems such as ATS and moving bed photobioreactors provide thermal and hydraulic stability that favors filamentous dominance [121]. Biofilm-based configurations also enhance biomass retention through the prevention of washout during periods of reduced growth. In some studies, wastewater heat recovery and greenhouse-integrated algal systems have been combined with filamentous cultivation to improve productivity under cold climates. These integrated approaches highlight the increasing convergence between environmental engineering and ecological process optimization [151]. Also, elevated ammonia concentrations can inhibit many microorganisms due to ammonia toxicity, pH imbalances, and free ammonia accumulation. Conventional nitrification systems treating high-ammonia streams often require extensive aeration and strict operational control, resulting in high energy consumption and operational costs [152].

But filamentous algae offer a biologically distinct pathway for ammonia management because they can directly assimilate ammonium as a preferred nitrogen source. Unlike nitrifying bacteria, which oxidize ammonia through energy-intensive autotrophic pathways, algae incorporate ammonium directly into amino acids and proteins through assimilatory metabolism. This process not only removes nitrogen but also converts it into harvestable biomass, thereby aligning with resource recovery objectives [153]. Several studies report that filamentous algae can tolerate and effectively remove ammonia concentrations significantly higher than those typically encountered in municipal wastewater systems [154]. The tolerance of filamentous algae to high ammonia is influenced by species-specific physiological traits and environmental conditions such as pH, temperature, and carbon availability. Filamentous algae such as *Oedogonium* sp. and *Cladophora* sp. have demonstrated strong ammonia uptake capacities in nutrient-rich wastewaters, particularly when sufficient inorganic carbon is available to sustain rapid growth. Their filamentous morphology may also contribute to ammonia tolerance by facilitating internal diffusion gradients and reducing direct exposure of all cells to toxic free ammonia concentrations [155]. One emerging insight is the importance of algal–bacterial interactions in stabilizing high-ammonia systems. In mixed consortia, filamentous algae assimilate ammonium while simultaneously producing oxygen that supports nitrifying and heterotrophic bacterial communities. This creates complementary nitrogen transformation pathways that improve overall treatment efficiency and reduce ammonia inhibition risks [156].

Hydraulic retention time (HRT) and nitrogen loading rate are key operational parameters governing the performance of filamentous algae-based wastewater treatment systems [157]. Reported HRTs generally range from approximately 2 to 10 days depending on reactor configuration, wastewater characteristics, temperature, and treatment objectives [158]. Shorter HRTs of approximately 2–3 days are often achievable in warm climates or highly productive attached-growth systems, whereas longer retention times of typically 5 days or more are commonly required under cold conditions or when treating high-strength ammonium wastewaters because reduced algal growth and nitrogen uptake slow treatment performance [159]. Also, attached-growth filamentous algal systems have demonstrated effective nitrogen removal at areal nitrogen loading rates of approximately 1–5 g N m^−2^ day^−1^, while optimized systems such as algal turf scrubbers have reported successful operation at loading rates approaching 10 g N m^−2^ day^−1^ [160]. Excessive ammonia loading can overwhelm algal assimilation capacity and lead to growth inhibition or biomass decay. However, attached-growth filamentous systems demonstrate improved resilience because high biomass retention enables sustained nutrient uptake even under fluctuating loading conditions. As a precaution, high-ammonia loading should be balanced with sufficient algal biomass to avoid free-ammonia toxicity and maintain stable nutrient removal. Increasing the biomass density generally improves ammonia tolerance because rapidly growing algae assimilate ammonium more quickly, thereby reducing the accumulation of inhibitory free ammonia. Operational loading rates should therefore be optimized according to biomass concentration, pH, temperature, and species-specific tolerance rather than a fixed ammonia-loading-to-biomass ratio [161,162]. In moving bed and ATS configurations, continuous harvesting further supports ammonia removal by maintaining active growth phases and preventing nutrient saturation within the biomass [163]. Another important consideration is pH dynamics because photosynthetic carbon uptake increases pH, which can shift ammonium equilibrium toward free ammonia (NH_3_), a more toxic form. While elevated pH may enhance phosphorus precipitation, excessive free ammonia accumulation can inhibit both algae and bacteria. Consequently, balancing the carbon supply, hydraulic conditions, and photosynthetic intensity is essential for maintaining stable operation in high-ammonia systems [164].

## 4. Resource Recovery from Filamentous Microalgae Used for Wastewater Treatment

Resource recovery from filamentous microalgae used in wastewater treatment is an emerging but sustainable approach that simultaneously addresses environmental pollution, renewable material production, and circular bioeconomy goals [165]. Conventional wastewater treatment systems are often energy-intensive and generate large amounts of sludge with limited reuse potential. This limited reuse stems from several factors because sewage sludge commonly contains heavy metals, pathogens, and persistent organic contaminants that restrict its beneficial use, while its high moisture content (typically 95–99%) makes dewatering one of the most energy-intensive and costly stages of sludge management. In addition, regulatory frameworks governing agricultural land application impose limits on contaminant concentrations to reduce risks of environmental pollution and contaminant transfer through food chains [166,167]. In contrast, filamentous algal biomass offers several advantages for resource recovery. It is naturally enriched in valuable biochemical components, including carbohydrates, proteins, and lipids, and its macroscopic filamentous morphology facilitates harvesting by simple screening or filtration, substantially reducing harvesting and dewatering costs compared with suspended microalgae [168]. Nevertheless, because filamentous algae can accumulate heavy metals and other contaminants during wastewater treatment, the suitability of harvested biomass for downstream applications should be evaluated according to the quality of the treated wastewater and the intended end use.

Sustainable resource recovery methods therefore transform wastewater treatment plants from waste disposal facilities into resource-generating biorefineries [169]. One of the most promising applications of filamentous microalgal biomass is cellulose extraction for bioplastics and paper production [170]. Filamentous green algae, including *Cladophora glomerata*, *Ulothrix* spp., and *Rhizoclonium* spp., possess cellulose-rich cell walls that represent a promising renewable source of high-quality cellulose. In particular, *Cladophora* cellulose exhibits exceptionally high crystallinity, while the absence of lignin simplifies cellulose extraction compared with terrestrial biomass. These characteristics make filamentous algal cellulose an attractive feedstock for cellulose-based bio composites, biodegradable films, specialty paper, membranes, and other high-value biomaterials [171,172].

Another important recovery pathway is biohydrogen generation through dark fermentation. After wastewater treatment, carbohydrate-rich algal biomass can be subjected to anaerobic fermentation by specialized microorganisms that convert organic substrates into hydrogen gas [173]. Biohydrogen is considered a clean and renewable energy carrier because its combustion produces only water. Filamentous microalgae are particularly suitable for this process due to their rapid growth and high organic content. Integrating dark fermentation with wastewater treatment offers dual benefits: renewable energy production and reduction in residual biomass disposal problems [174]. Despite its considerable potential, biological hydrogen production continues to face significant technical and economic barriers that limit large-scale commercialization. In dark fermentation, practical hydrogen yields are typically 1–3 mol H_2_ per mol glucose, considerably lower than the theoretical maximum of 4 mol H_2_ per mol glucose due to metabolic constraints and competing biochemical pathways. Higher theoretical yields (up to 12 mol H_2_ per mol glucose) are achievable only when dark fermentation is integrated with photo fermentation, allowing further conversion of fermentation by-products into hydrogen [175,176]. In addition, efficient separation and purification of hydrogen from carbon dioxide and other fermentation gases remain major process challenges that increase operational complexity and cost. The requirement for large-scale fermentation or photobioreactor systems also represents a significant obstacle to commercial implementation because of high capital investment and operational demands [177]. Furthermore, hydrogen storage and transportation remain major engineering challenges owing to hydrogen’s very low volumetric energy density, necessitating high-pressure compression or cryogenic liquefaction, both of which require substantial energy input and specialized infrastructure [178].

Filamentous microalgal biomass also has considerable value as a soil amendment and slow-release fertilizer. During wastewater treatment, algae assimilate essential nutrients, including nitrogen, phosphorus, potassium, and trace minerals. When processed and applied to agricultural soils, the biomass gradually releases these nutrients, improving soil fertility and reducing nutrient leaching compared to synthetic fertilizers. Additionally, algal organic matter enhances the soil structure, moisture retention, and microbial activity [8,179]. Despite these advantages, the widespread adoption of wastewater-derived algal fertilizers remains constrained by several technical, economic, and regulatory challenges. Freshly harvested algal biomass typically contains 85–95% moisture, so it needs to be dried to reduce transportation costs and increase shelf life. Drying or alternative stabilization processes is one of the most energy-intensive and costly stages of algae biomass processing [180]. In addition, wastewater-grown algae may accumulate heavy metals, pathogens, microplastics, and other contaminants, necessitating rigorous quality monitoring and appropriate post-treatment before agricultural application [181]. The chemical composition of algal biomass also varies considerably with wastewater characteristics, cultivation conditions, species, and season, making it difficult to produce standardized fertilizer products with consistent nutrient content and release characteristics [182]. Furthermore, regulatory approval pathways for wastewater-derived algal fertilizers remain limited or are still evolving in many jurisdictions, while concerns regarding product safety, quality consistency, and public acceptance continue to hinder broader commercialization. Filamentous algae are also a promising resource for aquafeeds because they are rich in proteins, essential amino acids, vitamins, pigments, and other bioactive compounds that can partially replace conventional feed ingredients and enhance fish growth and immunity. Their cultivation during wastewater treatment also supports resource recovery and contributes to a circular bioeconomy by converting waste nutrients into valuable biomass [183]. However, widespread application remains limited by the potential accumulation of heavy metals, pathogens, pharmaceuticals, and other contaminants in wastewater-grown biomass, requiring rigorous quality control and compliance with feed safety regulations. Additional challenges include variability in biomass composition, lack of standardized processing methods, evolving regulatory frameworks, and economic constraints associated with large-scale production [184].

### 4.1. Circularity of Bioplastics and Paper Production from Filamentous Algae Recovered from Wastewater Treatment

Among the most promising valorization pathways for wastewater-grown filamentous algae is the extraction and utilization of cellulose for cellulose-based bioplastics, bio composites, and specialty paper, extending resource recovery beyond nutrient recycling toward renewable material production [17]. Filamentous green algae such as *Cladophora glomerata*, *Spirogyra varians*, *Rhizoclonium riparium*, and *Oedogonium cardiacum* possess cellulose-rich cell walls that provide a renewable source of structural polysaccharides. Among these taxa, *Cladophora glomerata* has been particularly well studied because its cellulose exhibits exceptionally high crystallinity, high tensile strength, and a large specific surface area, making it an attractive feedstock for nanocellulose, packaging materials, membranes, specialty paper, and cellulose-based bio composites [185]. Compared with many unicellular microalgae, these filamentous species generally contain greater proportions of structural cellulose, facilitating cellulose recovery and downstream processing. When cultivated in wastewater treatment systems, filamentous algae simultaneously remove nutrients while generating cellulose-rich biomass, providing an integrated approach for wastewater remediation and renewable biomaterial production [186]. Algal cellulose offers several advantages over cellulose obtained from terrestrial lignocellulosic biomass. In particular, cellulose from *Cladophora glomerata* exhibits exceptionally high crystallinity, high tensile strength, and a highly ordered microfibrillar structure, while green algae contain little or no lignin, eliminating the need for the extensive delignification processes required for wood pulping [187]. Consequently, cellulose extraction from filamentous algae can be achieved by using comparatively milder chemical treatments, potentially reducing energy consumption, chemical usage, and environmental impacts. Recent studies have therefore investigated *Cladophora glomerata* as a promising source of nanocellulose for advanced materials. Owing to its excellent mechanical, barrier, and rheological properties, algal-derived nanocellulose has attracted considerable interest for applications including biodegradable packaging, biomedical materials, membranes, and reinforcement of biodegradable polymer composites [188].

The global accumulation of petroleum-based plastic waste has intensified the search for renewable and biodegradable alternatives [189]. Filamentous algal biomass represents a promising renewable feedstock for cellulose-based bioplastics and bio composites because it is biodegradable, reduces dependence on fossil resources, and has the potential to lower greenhouse gas emissions compared with conventional petroleum-derived plastics [190]. In particular, cellulose obtained from *Cladophora glomerata* possesses exceptionally high crystallinity and mechanical strength, enabling its use both as a reinforcing material and as a renewable polymer feedstock [191]. Cellulose derived from *Cladophora glomerata* has been successfully incorporated into biodegradable polymer matrices, including polylactic acid (PLA), starch-based polymers, and polyhydroxyalkanoates (PHAs), where it enhances tensile strength, thermal stability, and barrier properties [171,192]. These characteristics make algal cellulose an attractive material for biodegradable packaging, biomedical materials, and polymer composites. Furthermore, emerging biorefinery concepts propose the utilization of carbohydrates recovered from wastewater-grown algal biomass as fermentation substrates for microbial PHA production, thereby integrating wastewater treatment, biomass valorization, and biodegradable polymer production within a circular bioeconomy framework [170].

The paper and biomaterials industries represent another promising sector for the utilization of cellulose recovered from filamentous algae, thereby extending resource recovery beyond wastewater treatment [193]. In particular, cellulose isolated from *Cladophora glomerata* has attracted considerable attention as a renewable source of highly crystalline cellulose for specialty paper and advanced biomaterials [194]. The concept of industrial symbiosis has been proposed, in which wastewater-grown algal biomass is valorized as a supplementary cellulose feedstock, reducing waste generation while partially decreasing reliance on conventional lignocellulosic resources [195]. Owing to its nanoscale fibrillar structure, high crystallinity, and high specific surface area, nanocellulose derived from *Cladophora glomerata* has been investigated for specialty papers, filtration membranes, biodegradable packaging films, functional coatings, and polymer composites. These materials exhibit excellent mechanical strength and improved oxygen and moisture barrier properties, making them attractive for sustainable packaging and advanced engineering applications [187]. Consequently, the production of high-value materials such as nanocellulose and specialty cellulose products has been widely recognized as an effective strategy for improving the techno-economic feasibility of filamentous algae-based biorefineries [196].

### 4.2. Filamentous Microalgae Recovered from Wastewater as a Source of Protein for Aquafeeds

The increasing global demand for aquaculture products has intensified pressure on conventional feed resources, particularly fishmeal and soybean meal, which dominate modern aquafeed formulations [197]. This intense pressure for fishmeal production is attributed to declining wild fishing activities due to ecological concern. This increases the need for alternative and sustainable protein sources capable of supporting the rapid expansion of aquaculture without exacerbating environmental impacts caused by intense grain farming [198]. Filamentous microalgae cultivated in wastewater systems is an emerging tool for achieving this goal through a bio-circular economy, which simultaneously enables wastewater remediation, nutrient recovery, and protein-rich biomass generation [199]. Filamentous microalgae possess several characteristics that make them attractive candidates for aquafeeds. For example, Oedogonium, *Spirogyra*, *Cladophora*, and *Ulothrix* exhibit relatively high protein contents, favorable amino acid profiles, rapid growth rates, and the ability to assimilate nutrients directly from wastewater streams. Unlike conventional feed crops, these organisms do not require arable land or freshwater irrigation, and their cultivation contributes directly to nutrient removal from wastewater. This integration of biomass production with environmental remediation aligns strongly with circular economy principles, where waste-derived nutrients are reintegrated into food production systems [48]. Protein content in filamentous microalgae can vary widely depending on species, cultivation conditions, and wastewater composition [200,201]. Nitrogen-rich wastewaters, including aquaculture effluents, municipal side streams, and anaerobic digestates, often promote elevated protein accumulation due to enhanced ammonium availability [202]. Also, the amino acid composition of filamentous algae frequently includes essential amino acids required for fish growth, such as lysine, leucine, valine, and threonine. Although methionine levels may sometimes be limiting compared to fishmeal, strategic feed formulation and blending can compensate for such deficiencies [203]. The use of wastewater-grown filamentous algae in aquafeeds also contributes to nutrient circularity within aquatic food systems. Aquaculture itself generates nutrient-rich effluents containing nitrogen, phosphorus, and organic matter that can negatively impact receiving ecosystems if untreated [204]. The cultivation of filamentous algae on aquaculture wastewater creates closed-loop systems that reduce nutrient discharge and minimize dependency on external feed inputs and improve the overall sustainability of aquaculture operations. Recent studies have demonstrated promising performance outcomes when filamentous algal biomass is incorporated into aquafeeds [205]. The filamentous algae *Oedogonium intermedium* have received particular attention due to their favorable digestibility, nutritional composition, and supplementation for enhancing pigmentation, antioxidant status, and immune responses in cultured organisms, suggesting functional benefits beyond basic nutrition [206]. The biochemical composition of filamentous microalgae extends beyond protein and includes lipids, carbohydrates, vitamins, minerals, and bioactive compounds that may improve feed quality [207]. Pigments such as chlorophylls and carotenoids contribute antioxidant properties that can enhance coloration in ornamental fish and crustaceans [208]. Some filamentous species also contain polyunsaturated fatty acids (PUFAs), although generally at lower levels than certain marine microalgae. Additionally, bioactive polysaccharides and phenolic compounds present in algal biomass may exert antimicrobial or immunostimulatory effects, potentially improving disease resistance in aquaculture systems [209].

Despite their promising nutritional value and sustainability benefits, several limitations must be addressed before wastewater-grown filamentous algae can be widely adopted as aquafeed ingredients. Firstly, although filamentous algae are rich in protein, their digestibility is generally lower than that of fishmeal because cellulose-rich cell walls restrict the accessibility of intracellular proteins to digestive enzymes [210]. Digestibility varies among algal species, aquatic animal species, cultivation conditions, and processing methods. So, different approaches such as mechanical disruption (e.g., milling or homogenization), enzymatic hydrolysis (e.g., cellulase or protease treatment), fermentation, and extrusion processing have all been shown to improve protein availability by partially degrading cell wall structures and enhancing nutrient accessibility [211]. Thus, filamentous green algae such as *Cladophora glomerata* and *Oedogonium intermedium* have demonstrated potential as aquafeed ingredients, although their nutritional value and digestibility depend largely on biomass processing and cultivation conditions [206,212]. Another major concern for wastewater-derived algal biomass is the accumulation of heavy metals, including arsenic (As), cadmium (Cd), chromium (Cr), lead (Pb), and mercury (Hg), through biosorption and bioaccumulation. Elevated metal concentrations may exceed regulatory limits for animal feed, posing risks to animal health and food safety. Risk mitigation requires careful selection of wastewater sources, routine monitoring of biomass quality, compliance with feed safety standards, and, where appropriate, pretreatment methods such as washing or mild chemical extraction to reduce surface-bound metals while minimizing nutrient losses [213,214]. Other challenges include variability in biomass composition due to fluctuations in wastewater characteristics and cultivation conditions; high harvesting, dewatering, and drying costs; and the lack of harmonized regulatory frameworks governing the use of wastewater-derived algal biomass in animal feeds. These constraints can be mitigated through standardized cultivation protocols, optimized post-harvest processing, integrated biorefinery approaches that improve overall process economics, and comprehensive quality assurance programs to ensure product safety and consistency [215,216].

### 4.3. Filamentous Algae Recovered from Wastewater as a Source of Soil Amendment and Slow-Release Fertilizer

Filamentous algae not only remove nitrogen, phosphorus, and other contaminants from wastewater but also convert these dissolved nutrients into biomass rich in organic carbon, minerals, and bioactive compounds. This dual functionality positions them as important biological intermediaries connecting wastewater remediation with sustainable agriculture and soil restoration. Modern agriculture remains heavily dependent on synthetic fertilizers, particularly nitrogen- and phosphorus-based inputs derived from fossil fuels and finite phosphate reserves. Although these fertilizers have contributed significantly to global food production, their excessive use has generated severe environmental consequences, including eutrophication, greenhouse gas emissions, soil degradation, and nutrient runoff. Also, the wastewater streams produced during synthetic fertilizer treatment contain substantial quantities of recoverable nutrients that are often lost during conventional treatment processes. Filamentous algae offer a mechanism for capturing these nutrients biologically and returning them to agricultural systems in a more sustainable and circular form. Filamentous algal biomass generated from wastewater treatment is particularly suitable for agricultural application because of its favorable biochemical composition. Filamentous algae such as *Cladophora* sp., *Spirogyra* sp., *Oedogonium* sp., and *Rhizoclonium* sp. accumulate considerable amounts of nitrogen, phosphorus, potassium, calcium, magnesium, and micronutrients during wastewater cultivation. In addition to mineral nutrients, the biomass contains organic matter, amino acids, carbohydrates, vitamins, and plant growth-promoting compounds that can improve soil quality and crop productivity. Unlike soluble synthetic fertilizers that release nutrients rapidly and are prone to leaching losses, algal biomass generally decomposes gradually in soil, enabling more controlled nutrient release [217]. This slow-release characteristic is one of the most significant advantages of filamentous algae-derived fertilizers. Nutrients within algal biomass are primarily bound within organic cellular structures and therefore require microbial mineralization before becoming fully available to plants. This gradual decomposition process reduces nutrient losses through leaching, volatilization, and runoff, thereby improving nutrient use efficiency and minimizing environmental pollution. Jimenez et al. [218] report that soils amended with algal biomass exhibit sustained nitrogen and phosphorus availability over longer periods compared to conventional synthetic fertilizers [218]. The cellulose-rich and fibrous structure of filamentous algae also contributes positively to soil physical properties when incorporated into soil because they promote soil aggregation by acting as a “glue” and physical binder while also improving water-holding capacity and aeration. Filamentous algae also play a critical role in soil amendments because they support beneficial soil microbiota. This is because they provide a readily biodegradable carbon source that stimulates microbial growth and enzymatic activity in the rhizosphere [219]. Application of algal extract has been reported to stimulate beneficial soil microbial communities, including nitrogen-fixing bacteria, phosphate-solubilizing microorganisms, and arbuscular mycorrhizal fungi, thereby enhancing nutrient cycling, improving nutrient availability, and promoting plant growth [220,221]. In addition to bulk nutrient supply, filamentous algae contain various bioactive compounds that can promote plant growth and stress tolerance. Phytohormones such as auxins, cytokinins, and gibberellins have been identified in certain algal species and may stimulate seed germination, root development, and biomass accumulation [222]. Antioxidants, polysaccharides, and micronutrients present in algal biomass may also enhance plants’ resistance to abiotic stresses such as salinity, drought, and heavy metal toxicity. Consequently, wastewater-derived filamentous algae are increasingly being explored not only as fertilizers but also as bio stimulants within sustainable agriculture systems [223]. Recent research has also focused on converting filamentous algal biomass into more advanced fertilizer formulations through processes such as palletization, composting, hydrothermal treatment, and biochar production [224]. Pelletized algal fertilizers offer practical advantages in storage and field application, while co-composting algae with agricultural residues can enhance nutrient balance and organic matter quality. Similarly, algae-derived biochar may improve nutrient retention and soil carbon sequestration while reducing greenhouse gas emissions from soils [225]. The integration of filamentous algae into agricultural nutrient cycles is particularly relevant within the framework of wastewater circularity. Wastewater-derived nutrients traditionally regarded as pollutants are transformed into agricultural resources, thereby closing nutrient loops between the urban, industrial, and agricultural sectors. This approach reduces reliance on energy-intensive fertilizer production and mitigates nutrient discharge into aquatic ecosystems. In essence, filamentous algae serve as biological vectors for nutrient recirculation within circular bioeconomy systems [226].

### 4.4. Dark Fermentation of Filamentous Algae Biomass Recovered from Wastewater as a Source of Biohydrogen

Dark fermentation is an anaerobic microbial conversion of organic substrates into hydrogen, volatile fatty acids, and other intermediate metabolites in the absence of light. This process differs from photo fermentation or direct photolysis because it is not dependent on solar irradiation and can therefore operate continuously under controlled reactor conditions [227]. The process is typically mediated by fermentative bacteria such as *Clostridium* sp., *Enterobacter* sp., and *Bacillus* sp. that metabolize carbohydrates into hydrogen and organic acids. The integration of this process with wastewater-grown filamentous algae creates a synergistic system where nutrients are first recovered biologically during wastewater treatment and subsequently transformed into renewable energy carriers [228]. This is because filamentous algae such as *Spirogyra* sp., *Cladophora* sp., *Oedogonium* sp., and *Rhizoclonium* sp. accumulate substantial amounts of cellulose, hemicellulose, and storage polysaccharides under nutrient-rich wastewater conditions. These carbohydrates provide fermentable substrates for hydrogen-producing bacteria [229]. Importantly, the absence or minimal presence of lignin in algal biomass significantly reduces the recalcitrance commonly associated with plant biomass conversion, thereby lowering pretreatment severity and improving biodegradability [230]. The cultivation of filamentous algae in wastewater serves a dual purpose of treating wastewater and recovering nutrients for biomass production, thereby enhancing wastewater circularity while simultaneously influencing the biochemical composition of the biomass. The latter is critical because carbohydrate-rich biomass generally yields higher hydrogen outputs during dark fermentation, as carbohydrates are readily metabolized by fermentative bacteria [231]. For example, nitrogen limitation may enhance carbohydrate accumulation while reducing protein synthesis, whereas ammonia-rich wastewaters often promote protein-rich biomass, which may lower hydrogen productivity due to ammonia release during fermentation. Consequently, optimization of cultivation conditions is essential for tailoring biomass composition toward efficient hydrogen production [232].

Although filamentous algae are generally less recalcitrant than terrestrial lignocellulosic biomass because they lack lignin, their cellulose-rich cell walls still restrict microbial access to intracellular carbohydrates. Consequently, pretreatment methods such as thermal, acid, alkaline, enzymatic, ultrasonic, and mechanical treatments are commonly employed to disrupt cell wall structures and improve hydrolysis efficiency prior to dark fermentation [233]. Therefore, the integration of filamentous algal biomass recovered from wastewater treatment into dark fermentation provides an opportunity to recover additional value from the harvested biomass rather than treating it as a waste product. Furthermore, the fermentation effluent, which remains rich in volatile fatty acids and nutrients, can be further utilized for methane production through anaerobic digestion or recycled to support subsequent algal cultivation. This cascading resource recovery improves the overall biomass utilization and supports the development of integrated wastewater biorefineries based on circular economy principles [234]. Recent studies have also highlighted the importance of microbial consortia in improving fermentation stability and hydrogen productivity. Mixed bacterial communities often exhibit greater resilience to substrate variability and inhibitory compounds compared to pure cultures. In some systems, co-fermentation of filamentous algae with food waste, sludge, or agricultural residues has demonstrated synergistic effects that improve carbon balance, buffer capacity, and hydrogen yield. These integrated approaches are particularly attractive for municipal wastewater treatment facilities seeking multifunctional waste valorization strategies [6]. Beyond energy recovery, dark fermentation of filamentous algal biomass offers environmental benefits linked to greenhouse gas mitigation. Hydrogen produced biologically from wastewater-derived biomass represents a low-carbon energy carrier that can partially offset fossil fuel consumption. Moreover, algae-based wastewater treatment systems contribute to carbon capture through photosynthetic CO_2_ assimilation, creating opportunities for partially carbon-neutral or carbon-negative treatment pathways. Reduced sludge generation and nutrient recycling further enhance environmental sustainability compared to conventional wastewater treatment processes [235].

## 5. Limitation and Future Research Direction

Despite the growing body of evidence supporting filamentous algae as a multifunctional platform for wastewater treatment and resource recovery, several interconnected scientific, technological, economic, and regulatory challenges continue to limit their large-scale implementation and long-term operational sustainability [236]. One of the most significant limitations is the predominance of laboratory-scale studies performed under controlled conditions using synthetic or simplified wastewater. Although these studies have substantially improved the understanding of nutrient removal mechanisms and algal physiology, they often fail to capture the complexity of real wastewater systems, where fluctuations in wastewater composition, hydraulic loading, temperature, salinity, light availability, and microbial communities strongly influence treatment performance. Consequently, laboratory findings frequently cannot be directly translated into full-scale operation. Future research should therefore prioritize long-term pilot- and demonstration-scale studies using real wastewater under realistic environmental conditions, including seasonal and climatic variability, to establish reliable operational datasets and design criteria. Emerging digital twin technology may further support this transition by integrating process models with real-time operational data to evaluate different operational scenarios and optimize system performance before full-scale implementation [237]. Another important limitation is the inadequate taxonomic characterization of filamentous algal communities. Numerous studies continue to identify algae only to the genus level or describe mixed filamentous communities without molecular confirmation, despite considerable interspecific variation in nutrient uptake, stress tolerance, biomass composition, and resource recovery potential. Future investigations should integrate molecular taxonomy with genomic, transcriptomic, proteomic, and metabolomic approaches to identify species- and strain-specific functional traits associated with wastewater treatment and biomass valorization. This would facilitate the selection of strains best suited for particular wastewater types and climatic conditions. In the longer term, synthetic biology and metabolic engineering may provide opportunities to enhance desirable characteristics such as nutrient assimilation, stress tolerance, or biomass productivity, although these approaches remain largely at the experimental stage for filamentous wastewater algae [20,238].

Maintaining stable long-term performance is a major operational challenge, particularly in open and semi-open cultivation systems such as algal turf scrubbers and attached-growth reactors. Environmental fluctuations can promote biofilm detachment, self-shading, grazing pressure, and shifts in microbial community composition, reducing treatment efficiency and biomass productivity. Although filamentous morphology provides greater biomass retention than suspended microalgae, sustained operational stability under variable field conditions remains insufficiently understood. Future research should therefore focus on adaptive operational strategies, ecological engineering, and predictive process models capable of maintaining stable algal communities under changing environmental conditions. Artificial intelligence and machine learning have recently emerged as promising tools for analyzing operational datasets and supporting predictive process control, although their application to filamentous algal wastewater treatment systems is still at an early stage [239]. The ecological interactions among filamentous algae, bacteria, fungi, and other microorganisms are also poorly understood despite increasing evidence that these interactions strongly influence nutrient cycling, contaminant degradation, oxygen transfer, and biofilm stability. Current knowledge of metabolic cross-feeding, inter-kingdom signaling, microbial succession, and community resilience under dynamic wastewater conditions remains limited. Future studies should integrate metagenomics, meta transcriptomics, metabolomics, and systems biology to elucidate these complex microbial networks and support the rational design of stable and functionally optimized algal–microbial consortia [240,241].

Downstream processing is one of the principal economic barriers to commercialization because fresh algal biomass contains high moisture levels, making harvesting, dewatering, drying, and stabilization energy-intensive. In addition, wastewater-derived biomass may accumulate heavy metals, pharmaceutical residues, antibiotic resistance genes, microplastics, and pathogenic microorganisms, limiting its suitability for agricultural, aquacultural, or food-related applications without appropriate safety assessment. Future research should therefore prioritize standardized contaminant monitoring, biomass quality assessment, and the development of safe pretreatment technologies [242]. Regulatory uncertainty is also a major barrier to commercialization because wastewater-derived algal biomass is subject to different regulatory frameworks depending on its origin, processing method, and intended application. Requirements for contaminants, pathogens, and product safety vary considerably among jurisdictions, creating uncertainty for commercial developers. Future research should therefore generate robust toxicological, microbiological, and environmental safety data that can support science-based regulatory frameworks and standardized quality criteria. Close collaboration among researchers, regulators, industry, and end-users will be essential for developing practical approval pathways, improving public confidence, and facilitating market adoption. Harmonization of regulatory standards across jurisdictions would further support international commercialization [243].

Although numerous studies demonstrate the technical feasibility of filamentous algae for wastewater treatment, comparatively few incorporate comprehensive techno-economic analysis (TEA) and life-cycle assessment (LCA). Without these assessments, it remains difficult to determine whether proposed systems are economically competitive or environmentally sustainable at a commercial scale. Future research should integrate TEA and LCA from the early stages of process development to identify cost drivers, environmental hotspots, and opportunities for optimization. These analyses should consider regional variations in climate, wastewater composition, energy supply, infrastructure, and potential markets for algal-derived products to support informed technology development and policy decisions [244]. The challenges described above are closely interconnected and should not be addressed independently. For example, robust field-scale datasets are required to improve predictive process models, while improved taxonomic characterization can support strain selection for targeted resource recovery pathways. Likewise, advances in downstream processing will directly influence techno-economic performance and regulatory acceptance. Consequently, future research should adopt an integrated systems approach that combines pilot-scale validation, molecular characterization, advanced process monitoring, TEA/LCA, and sustainable biorefinery design. Emerging tools such as digital twins, artificial intelligence, and systems biology may further enhance decision-making and process optimization as these technologies mature. By integrating biological, engineering, economic, and regulatory perspectives, future research can accelerate the sustainable deployment of filamentous algae as multifunctional platforms for wastewater treatment and resource recovery [245].

## 6. Conclusions

Filamentous algae are increasingly recognized as multifunctional biological resources that are capable of transforming wastewater treatment from a pollutant-removal process into a platform for resource recovery and circular bioeconomy development. The collective evidence reviewed here demonstrates that these organisms can simultaneously support nutrient and contaminant removal while generating biomass that can be converted into bioenergy, biofertilizers, aquafeeds, cellulose-based biomaterials, and other value-added products. This multifunctionality distinguishes filamentous algae from many conventional treatment technologies and highlights their potential to integrate environmental remediation with sustainable production systems. Their ability to connect wastewater treatment, biomass valorization, and renewable material production illustrates how biological processes can contribute to more resource-efficient and resilient water management strategies. Although important scientific and engineering challenges remain, recent advances in cultivation systems, biomass processing, molecular characterization, and systems-level process integration have substantially expanded the opportunities for practical implementation. Continued progress will depend not only on technological innovation but also on closer integration of biological knowledge with engineering design, environmental policy, and circular economy principles. By positioning filamentous algae at the interface of wastewater management, biotechnology, and sustainable resource utilization, this emerging field has the potential to contribute meaningfully to future low-carbon, resource-efficient, and climate-resilient wastewater treatment systems.

## Figures and Tables

**Table 1:** Comparison of filamentous algal taxa for wastewater treatment.

Taxon	Taxonomic Group	Key Morphological Traits	Functional Traits and Wastewater Performance	Typical Wastewater Applications and Advantages	References
*Klebsormidium* sp.	Chlorophyta	Unbranched filaments with cylindrical cells	High biomass productivity; efficient N and P removal; reduces COD/BOD; tolerant to fluctuating conditions	Primary municipal and nutrient-rich wastewater; nutrient recovery	[6,44]
*Oedogonium* sp.	Chlorophyta	Unbranched filaments with characteristic cap cells	High biomass yield; efficient nitrate and phosphate removal; adaptable	Municipal, secondary, agricultural, and aquaculture wastewater	[45,46]
*Cladophora* sp.	Chlorophyta	Branched filaments with thick cell walls	Stable biomass; nutrient removal; high heavy metal biosorption.	Attached-growth systems; heavy-metal-contaminated wastewater	[47,48,49,50]
*Rhizoclonium* sp.	Chlorophyta	Thick, unbranched filaments	High biomass productivity; efficient nutrient uptake; tolerant to salinity and alkalinity	Municipal and saline wastewater	[6,51]
*Stigeoclonium* sp.	Chlorophyta	Irregularly branched filaments with strong rhizoids	Stable attached biofilms; efficient nutrient assimilation; shear tolerant	Algal turf scrubbers, biofilm reactors, stream remediation	[52,53]
*Spirogyra* sp.	Chlorophyta	Unbranched filaments with spiral chloroplasts.	Rapid N and P uptake; responsive to nutrient-rich conditions	Nutrient polishing and recovery from wastewater	[54,55]
*Oscillatoria* sp.	Cyanobacteria	Unbranched motile trichomes; non-heterocystous	Removes N, P, COD, heavy metals, and organic pollutants	Municipal, textile, agricultural, and industrial wastewater	[56,57]
*Anabaena* sp.	Cyanobacteria	Filaments with heterocysts and akinetes	P removal; nitrogen fixation; organic matter removal; high biomass production	Municipal and agricultural wastewater; biofertilizer production	[58,59,60]
*Nostoc* sp.	Cyanobacteria	Gelatinous filamentous colonies with heterocysts	P uptake; organic matter and heavy metal removal; stress tolerant	Municipal wastewater, agricultural runoff, metal-contaminated wastewater	[61,62,63]
*Phormidium* sp.	Cyanobacteria	Thin, unbranched filaments with mucilaginous sheath	Efficient nutrient removal; biofilm formation; tolerant to high organic loading	Municipal and industrial wastewater; biofilm reactors; attached-growth systems	[64,65]

## Data Availability

No new data were created or analyzed in this study. Data sharing is not applicable to this article.
